# The Effect of Replacement of Soybean Meal with Corn Dried Distillers Grains with Solubles (cDDGS) and Differentiation of Dietary Fat Sources on Pig Meat Quality and Fatty Acid Profile

**DOI:** 10.3390/ani11051277

**Published:** 2021-04-29

**Authors:** Małgorzata Świątkiewicz, Anna Olszewska, Eugeniusz R. Grela, Mirosław Tyra

**Affiliations:** 1 Department of Animal Nutrition and Feed Science, National Research Institute of Animal Production, Krakowska St. 1, 32-083 Balice, Poland; ankaol@poczta.onet.pl; 2 Institute of Animal Nutrition and Bromatology, University of Life Sciences in Lublin, Akademicka St. 13, 20-950 Lublin, Poland; eugeniusz.grela@up.lublin.pl; 3 Department of Pig Breeding, National Research Institute of Animal Production, Krakowska St. 1, 32-083 Balice, Poland; miroslaw.tyra@iz.edu.pl

**Keywords:** pigs, DDGS, dietary fats, meat quality and texture, fatty acid correlation

## Abstract

**Simple Summary:**

The growing demand for protein and the reluctance of consumers to use genetically modified feeds necessitate the use of other protein feeds. Corn dried distillers grains with solubles (cDDGS) is a well-digested protein feed; however, it is rich in unsaturated fatty acids and can negatively affect the meat quality and oxidative stability. The negative influence of dietary unsaturated fatty acids on meat quality can be balanced by feed additives, e.g., a dietary saturated fat source increasing the iodine value of fat. To reduce the detrimental effect of corn DDGS in the present experiment, the beef tallow and coconut oil in a feed mixture were studied, as both of them are more saturated than corn DDGS and rapeseed oil. The aim of the study was to investigate mixtures comprising corn DDGS as a partial replacer for soybean meal as well as different dietary saturated fat sources to determine their effect on the meat quality and fatty acid profile. The relationships between dietary fatty acid profile and meat fatty acid profile and between various meat quality parameters were analyzed.

**Abstract:**

The aim of the study was to investigate mixtures comprising corn distillers dried grain with solubles as a partial replacer for soybean meal (SBM) and different dietary fat sources, in order to determine their effect on the meat quality and fatty acid profile. Thirty-two crossbred fatteners were divided into four groups: I–SBM + rapeseed oil, II–cDDGS + rapeseed oil, III–cDDGS + beef tallow, IV–cDDGS + coconut oil. The experiment took place from 60 to 118 kg. At the end of fattening, all pigs were slaughtered and samples of meat (*musculus* *longissimus lumborum*) were taken. The fatty acid profile, texture, and quality traits were analyzed. Corn DDGS affected drip loss. Beef tallow and coconut oil improved water holding capacity and drip loss and increased fat content, compared to the control group. The dietary fat type affected the fatty acid composition, iodine value, and consequently some quality traits of meat. However, these relationships varied. Fat content in the meat was inversely correlated with shear force and texture parameters, but positively with tenderness and juiciness. The fatty acid profile significantly influenced cohesiveness, chewiness, resilience and sensory traits, which were the most beneficial in meat with higher fat content and higher fat saturation index.

## 1. Introduction

Soybean meal (SBM) is widely used in the nutrition of farm animals and is the basic protein feed used in the nutrition of intensively fattened pigs. It is a relatively expensive feedstuff, most often imported from Brazil and Argentina, where it is cultivated in vast areas, partially obtained by cutting down the rainforests. The high demand for protein increases the acreage of soybean crops at the expense of tropical forests, which disturbs the ecosystem and is associated with an unsustainable economy that has a negative impact on the global state of the environment [1,2]. In addition, soybean meal mainly comes from genetically modified plants, which arouses objections in some consumers. Replacing soybean meal with protein feeds of local origin, not GMOs, is a pro-consumer activity, but also protects the domestic feed market, and in several cases had no negative effects on pig performance [3,4,5,6]. Besides legume seeds and rapeseed, corn dried distillers grains with solubles (cDDGS) are a good solution for replacement of soybean meal. It is a well-digested protein feed with lower amount of anti-nutritional substances, unlike the other two sources, and should be used in animal feeding due to its high nutritional value [7]. DDGS is a popular feedstuff due to its increased availability and cost effectiveness when compared to other least-cost diet formulations.

However, the literature review indicates that DDGS can have a negative effect on fat firmness if used in high amounts [8]. The quality of meat and meat products must be taken into account, given the consumers’ requirements and the growing popularity of traditional pork products, for example long-maturing or dry-cured meats, ribs, and backfat-based products (bacon, smoked backfat). Dietary fat content and quality are two of the principal factors influencing the sensory and technological quality of meat, especially during the final fattening period. Corn DDGS is characterized by high content of unsaturated fatty acids (UFAs), which are prone to oxidation, and can have a negative effect on technological parameters, usefulness for processing, oxidative stability, and quality during storage [7,9]. Wang et al. [10] observed an increasing trend towards generation of thiobarbituric acid reactive substances (TBARS) when diets containing 15–30% of DDGS were administered, compared with corn–soybean meal-based diet. Furthermore, the oxidation process negatively affects meat quality traits, including the taste, flavor, and acceptability [11,12]. Especially, polyunsaturated fatty acids (PUFAs) are prone to oxidation and products of the oxidation thereof, i.e., pentanal and hexanal in the case of C18:2n-6 or 1-penten-3-ol and cis2-penten-1-ol alcohols in the case of C18:3n-3 can be detected in meat products and have been reported to deteriorate their consumer value [13]. UFA-rich meat and fat are characterized by reduced firmness, problems with separation of layers during slicing, and oily appearance in the package [14]. A high dietary amount of UFAs increases the concentration of these fatty acids in pork adipose tissues [15,16,17]. Oleic acid (C18:1n-9) prevails in the fatty acid composition of pig tissue (about 40%), but linoleic acid (C18:2n-6) shows the highest negative correlation with fat firmness and its technological traits [18]. Furthermore, the type of dietary fat not only has major effects on the fatty acid composition of raw meat and subcutaneous fat, but also significantly affects its suitability for processing and the quality of the final product [19]. In the case of pork, the characteristics related to the suitability for processing are particularly important, as about 80% of pork is used for sausages and the food industry and only about 20% is bought by consumers as raw meat. For example, in the UK, cured bacon and cooked ham only accounts for about one third of the total sales of pork products [19].

Early studies on fatty acid composition were mostly focused on adipose tissues, which is where the majority of fatty acids are located. Recently, more interest has been attracted by muscle tissues due to their greater significance as food. As shown by previous studies, the fatty acid composition in muscles is related to the consumer quality of meat, with saturated and monounsaturated fatty acids positively correlated with meat acceptance and polyunsaturated fatty acids correlated negatively [20]. The negative influence of dietary UFAs on the adipose tissue quality can be balanced by some feed additives, e.g., a saturated dietary fat source increases the iodine value of fat. To reduce the detrimental effect of corn DDGS in the present experiment, beef tallow and coconut oil in a feed mixture were studied, as both of them are characterized by more saturated fat than corn DDGS and rapeseed oil. The fatty acid profiles of these fats differ significantly, as described earlier [21]. In addition, coconut oil contains an element contributing to the health-promoting effect, i.e., lauric acid, which accounts for about 50% in its fatty acid profile. Medium-chain fatty acids are well known for their positive effect on the environment and the development of the gastrointestinal tract and the intestinal microflora, while lauric acid is particularly active against gram-positive bacteria, for example *Clostridium perfringens*, known as a frequent cause of diarrhea in pigs [22,23].

The research goal was the protection of processing quality and durability of the meat of pigs fed with feed mixture with a high content of cDDGS as a substitute for soybean meal. It was assumed that the addition of saturated fat to the feed mixture would balance the possible negative effects of unsaturated fatty acids on meat quality. The aim of the study was to investigate mixtures comprising corn DDGS as a partial replacer for soybean meal, as well as different dietary saturated fat sources, to determine their effect on meat quality and fatty acid profile.

## 2. Materials and Methods

### 2.1. Ethical Approval

All experimental procedures were approved by The Local Ethics Committee for Experiments with Animals in Cracow, Poland, and complied with the Directive 2007/526/EC of the European Parliament and the Council on the protection of animals used for scientific purposes. Throughout the experimental period, the health status of fattened pigs was regularly monitored by a veterinarian.

### 2.2. Animal Management, Diets, and Experimental Scheme

The experiment was carried out on 32 crossbred fatteners (50:50% gilts and barrows) originating from sows (Polish Landrace × White Large Polish) mated with a (Duroc × Pietrain) boar. The pigs were divided into four groups, with eight pigs in each: group I–SBM + rapeseed oil (RO), group II–cDDGS + rapeseed oil, group III–cDDGS + beef tallow (BT), group IV–cDDGS + coconut oil (CO). All diets were isonitrogenous and isoenergetic, and formulated to cover the nutritional requirements of the pigs [24]. The ingredient composition and nutritive value of the diets is shown in Table 1.

The experimental fattening lasted from 60 to 118 kg of body weight (BW). The fatteners were kept in individual straw-bedded pens and were fed individually with restricted feed amounts according to their body weight. The individual body weight of all fatteners was controlled every two weeks. The pigs were fed twice a day: 2.8 kg/d at 61–70 kg of BW, 3.0 kg/d at 71–80 kg of BW, and 3.2 kg/d at 80 kg of BW and heavier. During the trial, the animals had free access to water. At the end of the experiment, all pigs were slaughtered, carcass quality was evaluated according to standard methods used at the Pig Performance Testing Stations [25], and tissue samples were taken for analysis.

**Table 1:** Ingredient composition (g/kg) and nutritive value of diets used in the experiment.

Item	Group I (Control) SBM + RO	Group II cDDGS + RO	Group III cDDGS + BT	Group IV cDDGS + CO
Corn DDGS				
Barley	-	200.0	200.0	200.0
Wheat	435.6	417.1	417.1	417.1
Corn	250.0	250.0	250.0	250.0
Wheat bran	50.0	-	-	-
Soybean meal	50.0	-	-	-
Rapeseed oil	160.0	80.0	80.0	80.0
Beef tallow	30.0	30.0	-	-
Coconut oil	-	-	30.0	-
Vitamin-mineral premix *	-	-	-	30.0
NaCl	5.0	5.0	5.0	5.0
Calcium phosphate 1-Ca	2.3	2.1	2.1	2.1
Chalk	1.7	-	-	-
L-Lysine	13.0	12.0	12.0	12.0
DL-Methionine	1.8	3.3	3.3	3.3
L-Threonine	0.3	-	-	-
L-Tryptophan	0.3	0.2	0.2	0.2
	-	0.3	0.3	0.3
Content in 1 kg of feed mixture:				
ME ** (MJ/kg)	13.4	13.4	13.3	13.4
Dry matter (g/kg)	880	883	881	883
Crude protein (g/kg)	162	165	164	164
Crude fiber (g/kg)	39	42	42	42
Crude fat (g/kg)	50	68	66	68
Crude ash (g/kg)	45	42	43	43
Starch (g/kg)	425	372	372	372
Lys (g/kg)	9.0	9.1	9.1	9.1
Met + Cys (g/kg)	5.8	5.9	5.9	5.9
Tre (g/kg)	5.8	5.9	5.8	5.8
Trp (g/kg)	1.9	1.9	1.9	1.9
Phosphorus (g/kg)	4.5	4.5	4.5	4.5
Calcium (g/kg)	7.6	7.6	7.6	7.6

* Premix (per 1kg of premix): vitamin A, 1 300,000 IU; vitamin D3, 300,000 IU; vitamin E, 6000 IU; vitaminB1, 200 mg; vitaminB2, 600 mg; vitaminB6, 400 mg; vitamin B12, 6000 mcg; vitamin K, 300 mg; biotin, 2000 mcg; niacin, 4000 mg; folic acid, 50 mg; pantothenic acid, 1840 mg; choline chloride, 6912 mg; betainę 8000 mg; Cu, 4000 mg; Fe, 20,000 mg; I, 100 mg; Mn, 8000 mg; Se, 60 mg; Zn, 17,000 mg; Ca, 309 g; Cl, 1.835 g; K, 0.035 g; Mg, 30 g; Na, 0.037 g; S, 14.096 g. Amino acids content: Lys HCL-min. 98.5%; Met–min. 99%; Tre–min. 98.5%; Trp–min. 98%. ** ME-Metabolizable energy according to the equation proposed by Hoffmann and Schiemann [26].

### 2.3. Meat Sample Collection and Analysis

After 24h of carcass cooling at +4 °C, samples of meat (longissimus m.) for analysis were taken from the area between the last thoracic and the first lumbar vertebrae.

The acidity of the meat was controlled with a pH-meter equipped with a Metron OSH 12-00 electrode: 45 min after slaughter, after 24 h of cooling at +4 °C, and after 4 months of frozen storage at −20 °C.

Basic chemical analyses (dry matter, crude protein, crude fat) of meat samples were performed according to standard methods [27].

The fatty acid profile of the meat was estimated using gas chromatography with extraction of lipids from tissues according to the methods of Folch et al. [28]. The fatty acid profile was determined on a CP-Wax 58 capillary column (Varian BV, Middelburg, the Netherlands; 25 m, 0.53 mm, d.f. = 1 m, carrier gas = helium, 6 mL/min), with a column oven temperature program from 90 to 200 °C, using a Varian 3400 gas chromatograph (Varian Associates Inc., Walnut Creek, CA, USA) equipped with a Varian 8200 CX Autosampler (200 °C), FID detector (260 °C), and Star Chromatography Workstation software. All the analyses were performed in duplicate and mean values are given. The determined fatty acids are expressed in g per 100 g of all determined acids.

Thiobarbituric acid reactive substances (TBARS) were analyzed in the meat samples after 4 months of storage at −18 °C using a modified method proposed by Pikul et al. [29]. Briefly, 10 g of comminuted sample was homogenised with 50 mL of 4% perchloric acid with butylated hydroxytoluene (BTH) additive. After filtering, 5 mL of the filtrate was mixed with 5 mL of 2-thiobarbituric acid (0.02 M). The solution was heated in a sealed tube in a boiling water bath for 1 h, then cooled under running cold water for 10 min. The measurement was carried out at 532 nm against a calibration curve including a blank sample.

The percentages of individual fatty acids were used to calculate the iodine value (IV) of fat according to the following equation [30]:

IV = (16:1) × 0.95 + ([C18:1) × 0.86 + (C18:2) × 1.732 + (C18:3) × 2.616 + (C20:1) × 0.785 + (C22:1) × 0.723

The color of the meat was assessed with the CIE L* a* b* system using a Minolta CR-310 colorimeter. Brightness (L*), color saturation towards red (a*), and saturation towards yellow (b*) were measured in fresh samples (24h after slaughter). Then, the meat samples were frozen to the temperature of −20 °C and the meat color was examined again after storage for 4 months. The psychometric color saturation (C) and color change during storage (∆E) were calculated according to MacDougall [31]:

C = (a^2^ + b^2^)^0.5^

ΔE = [(ΔL)^2^ + (Δa)^2^ + (Δb)^2^]^0.5^

Samples of the longissimus m. were used for the analysis of meat Warner-Bratzler shear force. The meat samples were stored frozen (−20 °C) until the analyses were performed. The meat (200 g) was cooked to an internal temperature of +80 °C. After cooling for 45 min at room temperature, samples were cut parallel to the muscle fibers in the form of cylinders 15 mm in diameter and 15 mm high. The meat shear force was measured using a TA.XT Plus texturometer from Stable Micro Systems (Vienna Cort, Lampas Road, Godalming, Surrey GU7 1JG, England) with a Warner-Bratzler attachment fitted with a triangular cutout knife. During the test, the knife speed was 4.5 mm/s. The shear force is expressed as Fmax at the highest point of the cut curve (N). The cutting energy is presented as the value of the force acting on the cross-sectional area (N/cm^2^/s). Texture Profile Analysis (TPA) was carried out using the same texturometer with an adapter, which was a cylinder with a diameter of 50 mm. A test of double compression of the samples to 70% deformation of their height was performed. The speed of the roller was 2 mm/s, the pressure interval was 3 s, and the test detection threshold was 5 g. The analysis of the texture profile took into account such parameters as hardness, springiness, cohesiveness, chewiness, and resilience.

The water holding capacity of the meat was measured according to Grau and Hamm [32]. In order to assess the amount of meat weight loss during cooking, a thermal drip loss analysis was performed [33]. The meat samples were boiled in bags until the internal temperature reached +75 °C and then cooled. Meat weight loss during cooking was calculated according to the following formula:

Thermal drip loss (%) = ((sample mass before cooking–sample mass after cooking) × 100)/sample mass before cooking

The evaluation of meat sensory traits was made on a 5-point scale (1 = the poorest, 5 = the best) according to the methodology developed by Baryłko-Pikielna [34]. The samples were cut into about 30mm thick slices and boiled in a 0.6% NaCl water solution to an internal temperature +80 °C. After cooking, the meat was left at room temperature for 2 min to equalize the temperature in the sample. Then, the slices were cut into smaller pieces and presented to a panel consisting of six experienced persons. The aroma, taste, tenderness, and juiciness of the meat were evaluated.

### 2.4. Statistical Analysis

Statistical analyses of the treatment effect on the meat quality, texture profile, sensory traits, and fatty acid profile were conducted by one-way analysis of variance. The comparison of means was performed using Duncan’s multiple range test at a *p* ≤ 0.05 level of significance. All analyses of variance and calculation of Pearson correlation coefficients (r) were conducted using the Statistica 12 package (Copyright©StatSoft, Inc. 1984–2014).

## 3. Results

### 3.1. Fattening Results

The effect of the partial replacement of soybean meal with cDDGS, together with the various dietary fat sources, on weight gains and feed utilization in pigs was observed during the trial (60–118 kg BW) and was published elsewhere [21]. Briefly, the average daily weight gains in the three experimental groups ranged from 991 to 958 g, and feed utilization was from 3.0 to 3.2 kg; however, in comparison to the control group (986 g and 3.07 kg, respectively), these differences were not significant. Similarly, the parameters of carcass quality, i.e., cold dressing yield, meatiness, primal cut weight, and backfat thickness, did not differ significantly among the groups. The partial replacement of soybean meal with corn DDGS did not influence the fattening results.

### 3.2. Fatty Acids Profile of Meat

The analysis of the fatty acid profile in the meat confirmed the higher content of unsaturated fatty acids in the meat of pigs fed with corn DDGS with rapeseed oil, compared to the control animals obtaining soybean meal, mostly due to the MUFA and PUFA n-6 increase (Table 2). The content of C18:2n-6 was higher by 8% and the iodine value was higher by 4.5% in the corn DDGS with rapeseed oil group (*p* > 0.05), compared to the control group without corn DDGS supplementation. The addition of beef tallow or coconut oil significantly increased the fat saturation index of the meat and decreased the iodine value in comparison to group II receiving rapeseed oil (*p* ≤ 0.05). The content of C18:0 was similar in all groups, while the amount of C16:0 was similar to that in the control group. The levels of PUFA n-3 were lower in the meat of pigs fed with beef tallow or coconut oil (*p* ≤ 0.05). However, the highest content of lauric acid was detected in the group receiving coconut oil (*p* ≤ 0.05). The content of C18:2n-6 in the meat was substantially lower in groups fed with saturated dietary fat (beef tallow, coconut oil), compared to that in the groups supplemented with rapeseed oil. Oleic acid, i.e., the most typical acid in the pork fatty acid profile, accounted for about 35–37% and was similar in all groups.

### 3.3. Correlations between Fatty Acid Content in the Diet and the Content of the Same Fatty Acids in Meat

**Figure 1:**
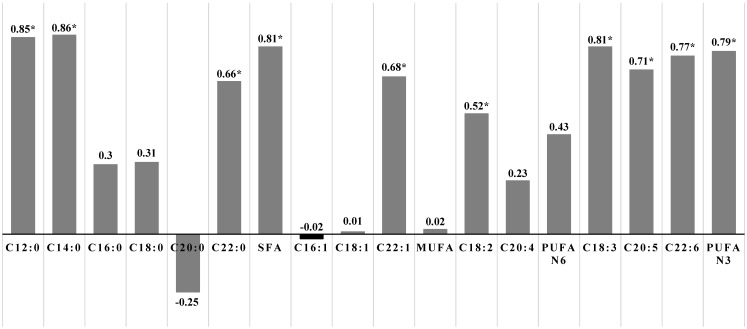
Correlation coefficients between fatty acid content in the diet and the content of the same fatty acids and the iodine value in meat (*longissimus* m.)**.** (* correlation statistically significant at *p* ≤ 0.05).

The correlations between the fatty acid content in the diet and the content of the same fatty acids in the meat (*longissimus* m.) are presented in Figure 1. It was observed that the dietary saturated fatty acids were positively correlated with these fatty acids in the meat (r = 0.7–0.9), including C12:0, C14:0, and C22:0 acids, but not C16:0 and C18:0. Dietary PUFA n-3 were also positively correlated with PUFA n-3 in the meat (r = 0.8; *p* ≤ 0.05). In the case of the sum of MUFA and PUFA n-6, the correlations between the feed and the meat were low and statistically not significant, except C22:1n-9 (r = 0.68; *p* ≤ 0.05) and C18:2n-6 (r = 0.52; *p* ≤ 0.05). The meat iodine value, indicating the unsaturation degree of its fat, was highly influenced by the dietary fatty acids (r amounted to 0.7–0.8).

### 3.4. Meat Quality Indices

The dietary treatment did not affect the meat acidity, neither in the fresh nor in the frozen stored samples, or the meat oxidative stability measured after 4 months of frozen storage (Table 3).

The basic chemical analysis of the meat showed significant differences (*p* ≤ 0.05) in protein and fat content (Table 3). The fat content was higher in the experimental groups, especially in those supplemented with beef tallow and coconut oil, where it was higher by about 70% in comparison to the control group.

The lowest values of water holding capacity measured in the fresh meat (18.80–18.65 cm^2^/g) was observed in the groups receiving beef or coconut fat in the feed mixture (Table 3). The water holding capacity of meat in these groups was about 16% higher than in the control group (*p* ≤ 0.05). A similar tendency was also observed after 4 months of frozen storage, as the lowest water absorption (13.8–13.9 cm^2^/g) was found in the meat of pigs from the two groups receiving mixtures with saturated fats. The differences were not statistically significant, but the values obtained in these groups were 11–12% higher than in the control group. Muscle samples were also cooked to assess the degree of weight loss during the heat treatment, i.e., thermal drip loss (Table 3). It was found that the meat of the control group lost the highest amounts of water (27%) and the largest percentage per mass, while the thermal drip loss in the experimental groups was approximately 24% of the meat sample weight (*p* > 0.05).

The color of fresh and frozen meat from the different diet groups was examined, and no statistically significant differences were found between the groups (Table 3). After 4 months of frozen storage, it was found that the color of meat in all groups was darker and more saturated towards red and yellow than meat measured 24 h after slaughter. The degree of color saturation (C) was slightly higher in the fresh meat (16.2–17.5) than in the frozen meat (17.1–17.8). The lowest color change during storage (ΔE) was observed in the control group, but the differences were not statistically significant.

### 3.5. Meat Shear Force and Texture Parameters

The meat shear force in all groups did not differ significantly (Table 4). However, it can be indicated that these values in the experimental groups were numerically lower than in the control group. In the meat of pigs receiving saturated fats (group III and IV), the shear force was lower by 7% and 9.2%, respectively, than in the control group (*p* > 0.05). Cohesiveness, chewiness, and resilience were also lower in these two groups (*p* ≤ 0.05).

### 3.6. Meat Sensory Indices

All the sensory features of cooked meat (Table 5) were rated highly and the scores ranged from 4.5 to 4.9 (1 to 5 scale). The aroma and taste did not differ significantly among the groups, but tenderness and juiciness were significantly better in the experimental groups than in the control (*p* ≤ 0.05).

### 3.7. Correlations between Groups of Fatty Acids in Meat and Meat Quality Traits

Table 6 presents the correlation coefficients between chosen fatty acids or groups of fatty acids in the meat and various meat quality parameters (texture, organoleptic traits). PUFAs were directly correlated with the shear force and texture parameters of cooked meat, which means that the higher PUFA contents are accompanied by undesirably higher values of these parameters. The shear force was significantly influenced by C18:2n-6 (r = 0.4; *p* ≤ 0.05), while most of the texture parameters (cohesiveness, resilience, springiness, and chewiness) were significantly influenced by C18:3n-3 (0.4 < r < 0.7; *p* ≤ 0.05). PUFAs were inversely correlated with the organoleptic traits, but a significant relationship was observed in the case of tenderness and juiciness with C18:3n-3 (r = 0.7; *p* ≤ 0.05). SFAs were inversely correlated with shear force and texture parameters and directly correlated with organoleptic traits, but a significant relationship was observed with cohesiveness and resilience only, especially in the case of C16:0 (r = 0.5; *p* ≤ 0.05). Palmitic acid did not have any significant effect on the organoleptic traits, while stearic acid had a much more positive impact on these traits, especially juiciness (r = 0.43; *p* ≤ 0.05). MUFAs were inversely correlated with texture parameters, including C18:1n-9, especially in the case of springiness (r = 0.46; *p* ≤ 0.05). The correlations of meat aroma and taste as well as water holding capacity with the fatty acid profile were very low (*p* > 0.05). The iodine value of the meat was directly correlated with shear force and texture parameters and inversely correlated with organoleptic traits. A significant correlation was observed between the iodine value and shear force (r = 0.41), cohesiveness (r = 0.56), resilience (r = 0.53), and juiciness (r= −0.41).

The fatty acid profile of meat appeared to be significantly correlated with fat content in the meat (Table 6). PUFAs were inversely correlated with fat content (r = 0.54 to 0.73), SFAs were directly correlated with this parameter (r = 0.43 to 0.73), and the correlation between MUFAs and fat content was not significant. The iodine value was negatively correlated with fat content (r = −0.52; *p* ≤ 0.05).

The meat color saturation (Table 6) measured 24h after slaughter was directly correlated with PUFAs n-3, including C18:3n-3 (r = 0.6; *p* ≤ 0.05), and a higher content of these acids significantly increased the meat redness. MUFAs (and C18:1n-9) were positively correlated with meat lightness (r = 0.5; *p* ≤ 0.05).

Meat pH measured 45 min after slaughter was directly correlated with MUFAs and C18:1n-9 (r = 0.5; *p* ≤ 0.05), while pH after 24h was not affected by any of the fatty acids (Table 6). Neither the water holding capacity after slaughter nor the drip loss of cooked meat was correlated with the fatty acid profile.

### 3.8. Correlations among the Chosen Quality Traits of Meat

**Figure 2:**
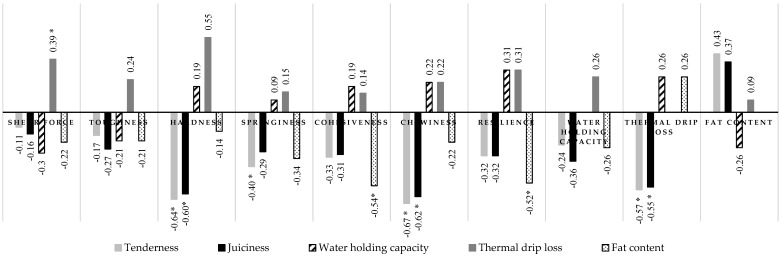
Correlation coefficients for chosen quality traits of meat (*longissimus* m.) (* correlation statistically significant at *p* ≤ 0.05)**.**

The correlations among chosen meat traits connected with consumer quality and texture, which are important for meat processing and finally affect product quality, were analyzed in the present experiment (Figure 2). Thermal drip loss was directly correlated with all meat texture parameters and, in the case of shear force, the correlation was significant (*p* ≤ 0.05). Inverse correlations between tenderness and juiciness in combination with all the estimated meat traits were observed, but the correlation coefficients were significant in the case of hardness, chewiness, and thermal drip loss (*p* ≤ 0.05). Water holding capacity was inversely correlated with shear force and toughness, and directly correlated with the other texture parameters and organoleptic traits; however, all the coefficients were low. A direct but low correlation was found between the water holding capacity of the meat after slaughter and the thermal drip loss (*p* > 0.05). The fat content in the meat was inversely correlated with all estimated meat quality traits, but a significant relationship was observed in the case of cohesiveness and resilience (r = −0.5; *p* ≤ 0.05). The fat content was positively correlated with tenderness and juiciness (r = 0.4; *p* > 0.05).

## 4. Discussion

The partial replacement of soybean meal with DDGS did not have any significant effect on weight gain, feed utilization, and carcass quality, as published elsewhere [21]. It can be concluded that all feed mixtures were well balanced in nutrients and energy concentration, and the partial replacement of soybean meal with corn DDGS, as well as the rapeseed oil replacement with beef tallow or coconut oil, did not influence the fattening results. It was observed earlier that no fattening indices and carcass traits were affected by the presence of corn DDGS in the feed mixture, even up to 30%, during the whole fattening cycle [10] or when pigs were fed 40%, 30%, 20%, and 10% of corn DDGS in fattening phase 1 to 4, respectively [35]. Similarly, Teye et al. [36] did not observe an effect of saturated dietary fats (palm kernel oil, palm oil) on body weight gains, feed conversion, and carcass quality.

Nutrition is a promising route for regulation of the meat fatty acid profile. In pigs, dietary fatty acids are incorporated directly into tissue lipids, unlike in cattle and sheep, in which dietary PUFAs are hydrogenated in the rumen. The most significant effects can be achieved using diets with high level of C18:2n-6, which is present in grains and oilseeds, and the proportion of this fatty acid in tissues increases linearly as the dietary intake increases [13]. The literature evidence suggests that more than 60% of the change in the fatty acid composition in porcine adipose tissue is caused by the dietary fat concentration or source, and its effect occurs after the first 25 days of feeding. In turn, in the case of muscle tissue (*longissimus* m.), the majority of changes in the fatty acid composition occur within 4 to 5 weeks [37]. An earlier study demonstrated that maximum incorporation of dietary C18:3n-3 in muscle tissue was achieved after 60 days on feed [38].

The methods of manipulating the fatty acid composition in meat still arouse considerable interest, mostly because saturated fatty acids are believed to be implicated in formation of blood clots leading to heart attack, cardiovascular diseases, and type 2 diabetes [39]. Most often, the goal of the manipulation is to improve the PUFA:SFA ratio. Some investigations are focused on the types of PUFAs and the lower n-6:n-3 PUFA ratio in the diet, as high amounts of PUFA n-3 are beneficial for health [20]. On the other hand, fatty acids are involved in various aspects of meat quality, shelf life, and processing technology, and polyunsaturated fatty acids are highly prone to oxidation. In view of the quality, durability, and consumer quality of meat, attention should be paid to the content of saturated fatty acids, which are associated with firmness of meat fat [20]. With their different melting points, fatty acids have an important effect on the firmness or softness of meat fat [40]. Groups of fat cells containing saturated fat with a high melting point appear whiter; hence, the color of fat is another aspect of meat/fat quality affected by fatty acids. The fat content may also affect the results of apparatus-assisted analyses of meat color. Increased levels of fat in the muscle tissue in the form of marbling will give a lighter color than the real one as the result of reflection of light from such clumps of fat, which is an achromatic substance. In addition, higher fat content in meat, especially unsaturated fat, can cause the so-called “film effect”, which also reflects part of the measuring light beam emitted by the apparatus, and a lighter color effect occurs. Human eyesight is not as easy to deceive, which is why color tests often include additional sensory analyses based on a color scale assessed by laboratory technicians. Several consumer acceptance studies have identified the color of meat as the most important trait considered at purchase [41]. The ability of unsaturated fatty acids to oxidize is important in regulating the shelf life of meat (rancidity and color deterioration). In an experiment on six pig genotypes, Zhang et al. [42] observed that an increase in MUFAs (C18:1n-9) and a decrease in SFAs (C16:0) tend to deteriorate fat deposition traits, IMF, and color scores, but exert positive effects on the moisture content of meat. In our experiment, the correlation analysis indicated that the higher MUFA (C18:1n-9) levels and the lower C18:2n-6 content in the fatty acid profile were accompanied by a lighter meat color (r = 0.5). The higher content of C18:3n-3 was significantly correlated with better saturation in red color (r = 0.6). In turn Teye et al. [36] reported a tendency for slightly higher redness and yellowness in the meat of pigs fed with palm kernel oil (*p* > 0.05) and significantly higher color saturation in comparison to the soybean oil treatment. Such an effect may have been linked to the higher concentrations of C12:0 and C14:0 in the meat, which makes the constituent lipid less translucent and therefore brightens the color.

In the experiment conducted by Zhang et al. [42], fatty acid composition showed the strongest significant correlations with fat deposition. Most remarkable was backfat thickness and fat content in meat, which displayed negative correlations with C18:2n-6 and PUFAs in six pig populations and positive correlations with C18:1n-9 in five pig populations. As shown by Kouba et al. [38], pigs with higher total lipid mass per 100g of muscle were characterized by higher content of C18:1n-9 and lower content of C18:2n-6 in the fatty acid profile. Wood et al. [13] reported dependence between the fatty acid profile and tissue fat, i.e., a negative relationship with C18:2n-6 and a positive correlation with C18:1n-9. Similarly, a negative correlation of the fat content in meat with PUFAs (C18:2n-6 and C18:3n-3) was observed in our experiment, but the positive correlation with MUFA (C18:1n-9) was not significant. The iodine value was also significantly correlated with the fat content in the meat, which means that a lower iodine value can be an indicator of higher fat content in meat, as shown in our experiment.

Dietary fats with different saturation degrees used in the present experiment affected the C18:2n-6 level in the meat; for example, the corn DDGS raised the C18:2n-6 amount in comparison to the control group (18 vs. 16%), and the correlation of this fatty acid in the diet and meat was significant. Dietary beef tallow and coconut oil decreased the C18:2n-6 content in the meat. It is known that, in pigs, linoleic acid is derived entirely from the diet and it is absorbed unchanged into the blood in the small intestine and incorporated into tissues [13]. The amount of C18:2n-6 observed in this study did not exceed the standard level (about 10–15% of all fatty acids), which is a beneficial effect, since muscles with highly elevated C18:2n-6 levels oxidize rapidly when heated, producing various volatile compounds, including aldehydes [40]. Similarly, C18:3n-3 was affected by the dietary fat sources, but its level did not exceed 1.5%, which is a positive phenomenon, as at a C18:3n-3 level above 3% of total fatty acids, the volatile compound production during thermal treatment can be enhanced and negatively influence meat flavor. The analysis of the fatty acid profile in the meat confirmed the higher content of UFAs and lower content of SFAs in the meat of pigs fed with corn DDGS with rapeseed oil (group II), compared to the control group fed soybean meal with rapeseed oil (group I). A similar effect was observed by Wang et al. [10] in pigs receiving 15% and, especially, 30% of corn DDGS. In the present study, the content of C18:2n-6 was higher by 8% and the iodine value was higher by 4.5% in the corn DDGS with rapeseed oil group, while in the experiment conducted by Wang et al. [10] C18:2n-6 was higher by 9% when 30% of corn DDGS was included in the feed. The authors observed higher TBARS in stored meat of pigs fed with corn DDGS, while this difference in our experiment was very low and insignificant. Lee et al. [43] observed significantly higher content of C18:2n-6 in meat; however, the total sum of unsaturated and saturated fatty acids did not differ. The results showed that the corn DDGS included in pigs’ diet significantly influenced the fatty acid profile in meat.

The effects of dietary fat of plant and animal origin incorporated into diets at 5% on the fatty acid composition of the *longissimus* m. in growing-finishing pigs was tested by Apple et al. [37]. In their experiment, the total sum of SFAs in the meat of pigs fed with 5% of beef tallow did not differ from that in pigs fed with soybean oil, but the sum of MUFAs and PUFAs was significantly higher. Due to the higher content of unsaturated fatty acids in the meat, the iodine value was over 8% higher in the meat of the soybean oil vs. beef tallow-fed pigs. In the present experiment, the content of SFAs and UFAs in the meat was significantly different between groups fed with the different dietary fat sources (rapeseed oil vs. beef tallow or coconut oil), whereas the sum of MUFAs was similar. The iodine value was lower by 10% when beef tallow was used, in comparison to rapeseed oil, which is in agreement with the findings reported by Averette Gatlin et al. [44], where the iodine value decreased in response to inclusion of 5% beef tallow in the finishing diet. The results of this study and other data demonstrated that iodine value in the muscle tissue could be altered by the dietary fat fed to growing-finishing pigs. Our experiment confirmed the significant correlation between the dietary SFA and PUFA n-3 contents and the amount of these acid groups in meat (r = 0.8) as well as the iodine value. The dietary fat iodine value appeared to be a good indicator of the fat saturation degree in meat. In the present experiment, the content of C18:2n-6 and C18:3n-3 in the group fed with rapeseed oil was significantly higher than in the groups fed with saturated dietary fat (beef tallow or coconut oil). Pigs do not synthesize C18:2n-6 and C18:3n-3; therefore, their content in meat is a reflection of these fatty acids in the diets [45]. However, the reduced content of C18:2n-6 and C18:3n-3 in the group fed with beef tallow is indicative of greater generation of SFAs and MUFAs via de novo synthesis replacing PUFAs [46].

Teye et al. [19,36] tested the effect of feed mixtures containing palm kernel oil (high in C12:0, C14:0, and C18:0 acids), palm oil (high in C16:0 and C16:1 acids), and soybean oil (high in C18:2n-6 acid). The greatest dietary impact was exerted on the proportions of C12:0, C14:0, and C18:2 in the muscle, while the C16:0 and C18:0 and MUFAs were hardly affected by dietary fatty acids. In our experiment, the SFA content in the meat was significantly correlated with dietary SFAs, but in the case of MUFAs the correlation was negligible. In our experiment the content of SFA in the control and coconut oil diet differed significantly (17% vs. 54%), as well as MUFA (44% vs. 16%) [21]; however, in meat the content of MUFA was very similar (control group 37.9% vs. coconut oil group 38.3%). These results are explained by the fact that C12:0 and C14:0 are mainly (but not entirely) derived from diet, whereas C16:0, C18:0, and MUFAs are mainly the products of synthesis in the animal, and this indicated that the interconversions between them limits the impact of dietary treatment [13]. In addition, the coconut oil, rich in SFA, used in the diet significantly improved the expression of the stearoyl-CoA desaturase gene (SCD) [21]. The enzyme SCD catalyzes the formation of double bonds between the ninth and tenth carbon atoms of certain SFAs and, therefore, is responsible for the conversion of these acids to MUFAs.

The absence of a significant effect of corn DDGS on meat acidity, color, shear force, and drip loss noted in the present experiment was in accordance with the results obtained by Wang et al. [10] and Harris et al. [35]. Similarly, there was no significant effect of 10% and 20% addition of corn DDGS to the finisher diet on meat acidity, color, cooking loss, and shear force in the experiment conducted by Lee et al. [43]. The authors observed higher content of crude fat in meat, but the difference was not significant, as in the present experiment (group I and II). The dietary fat (rapeseed oil, beef tallow, and coconut oil) did not affect the meat color in our experiment, but the saturated fats (beef tallow and coconut oil) significantly improved the water holding capacity and drip loss and increased the fat content in the meat, compared to the control group. The saturated dietary fat (palm oil) used in an experiment by Teye et al. [36] did not affect the meat color or drip loss. The color change may have been related to the oxidation of red oxymyoglobin to brown metmyoglobin in a reaction generally proceeding in parallel to that of fat rancidity. The lipid oxidation products can promote pigment oxidation and vice versa, although the strength of the relationship between these two aspects of shelf life is sometimes low [47]. In the present study, the color of meat after slaughter and after storage did not differ, which is supported by the fact that the levels of TBARS did not vary among the groups. In our experiment, the amount of TBARS measured after 4 months of frozen storage amounted to 0.46–0.49 mg/kg. Values above 0.5 are considered critical, as they indicate the presence of lipid oxidation products responsible for a rancid odor and taste detectable by consumers [13].

Parameters of technological meat quality that can be influenced by fatty acids, include firmness (hardness), shelf life (lipid and pigment oxidation), and flavor. In our study, the fatty acid profile in the meat was reflected in several texture parameters and sensory traits, which were the most beneficial in the meat with the highest fat content and a low PUFA:SFA ratio. In the experiment conducted by Teye et al. [36], various dietary fats affected the fatty acid profile of meat, but no significant differences were observed in the shear force and consumer value of the meat, except tenderness, which was best in meat characterized by the lowest PUFA:SFA ratio and the highest IMF content. The fat PUFA:SFA ratio is an indicator of the fat’s nutritional value in meat intended for human consumption. In our experiment, the use of saturated dietary fats (beef tallow and coconut oil) reduced the PUFA:SFA ratio in the meat, but its amount was not below 0.4, maintaining a safe level. However, these observations indicate that there may be limitations in the use of diets with saturated fat content higher than that in the present experiment for meat production.

It seems that meat tenderness and juiciness can be affected by the total amount of dietary fatty acids rather than the individual examples. The effect of fatty acids on meat firmness is related to the different melting points; an increase in fat saturation is accompanied by an increase in the melting point and firmness [40]. In the present experiment, the correlation of single acids with meat quality traits was similar to the correlation of the fatty acid groups (MUFA, PUFA n-6, PUFA n-3, SFA), but some differences were noticed. In terms of meat technological value and consumer quality, the content of SFAs is associated with firmness, while MUFAs are positively and C18:2n-6 is negatively correlated with flavor and overall acceptability of meat [20]. The correlation analysis carried out in the present study showed that the higher SFA content decreased the shear force of the meat, but this correlation was quite low. The meat acceptability itself was not measured, but the higher content of C18:1n-9 was correlated with the lower (better) values of texture parameters and better tenderness. The undesirable effect of C18:2n-6 and C18:3n-3 on meat texture and organoleptic traits was observed in the present experiment, but the aroma and taste were not shown to be significantly correlated with the fatty acid profile. Zhang et al. [42] observed a positive correlation of C16:0 with pH45 values of *longissimus* m. and a positive correlation of C18:2n-6 and PUFAs, as well as a negative correlation of C18:1n-9 and MUFAs with the moisture content in meat. In our experiment, the thermal drip loss, but not the water holding capacity, was positively correlated with PUFA n6. Cameron et al. [20] observed that C18:2, C18:3, C20:3, C20:4, C20:5, C22:5, and C22:6 were negatively correlated with meat flavor, flavor liking, and overall acceptability; however, fatty acids C16:1 and C18:1 were positively correlated with these consumption-related traits of meat.

Low intramuscular fat content in meat, which has a negative effect on marbling, may substantially deteriorate meat quality for consumers [36]. For example, selection of pigs for high lean growth reduced intramuscular fat content by 3 mg/g and was associated with poorer flavor [20]. In the present experiment, tenderness and juiciness obtained better scores in meat with higher crude fat content, fat with a higher saturation degree, and a lower iodine value (groups III and IV). The correlation between fat content and consumer quality of this meat was positive (r = 0.4). In addition, tenderness and juiciness were significantly negatively correlated with PUFA n-3 (r= −0.7), and the correlation analysis showed that a lower meat iodine value was associated with higher scores for tenderness and juiciness. Besides better tenderness and juiciness, better results for shear force and texture parameters in meat with higher content of crude fat were also found. As shown by Wood et al. [13], several studies confirmed the positive relationship between total lipid (marbling fat) of meat (*longissimus* m.) and its consumer quality, especially juiciness, which is associated with greater retention of water in meat during cooking. The results of a consumer acceptability study carried out by Font-i-Furnols et al. [41] showed that both “lean loin lovers” and “marbled loin lovers” gave increased palatability scores for cooked pork with increased intramuscular fat content. A minimum IMF content of 2.2–3.4% is recommended for good consumer acceptability. The positive effect of lipids on tenderness can be explained by the location of lipids in fat cells within the connective tissue perimysium, which may have a physical effect on separating muscle fiber bundles and beginning the process of tenderization by opening up the muscle structure. In addition, lipids can also trap moisture in muscle, thus improving juiciness and finally allowing muscle fibers to be more easily broken down in the mouth [40] as well as improving lubrication during chewing [41]. In the human perception of meat juiciness, the fat content in meat may have another effect, which is the so-called maintenance of the impression of juiciness by stimulating the salivary glands to produce saliva. It turns out that the maintenance of the feeling of juiciness while chewing leaves a longer lasting impression than the first feeling of fluid (native juiciness). Therefore, a higher correlation can be found between meat juiciness and fat content than between juiciness and the amount of liquid squeezed out of the meat during chewing (actual juiciness determined by the water absorption of the meat), as confirmed in our experiment. In the present study the aroma and taste of the meat was not affected by the dietary fat type or its saturation degree, although the fat tissue in meat is considered as the source of the characteristic flavors.

## 5. Conclusions

The partial replacement of soybean meal with corn DDGS and the replacement of rapeseed oil with beef tallow or coconut oil did not influence fattening results, since the feed mixtures were well balanced in nutrients and energy concentration. The corn DDGS did not exert a significant effect on the acidity, color, shear force, and drip loss of meat. Beef tallow and coconut oil significantly improved the water holding capacity and drip loss as well as increased the fat content in the meat, compared to the control group. The results have shown that dietary fat type affects the fatty acid composition of meat and iodine value. Consequently, the meat fatty acid profile influences some of its quality traits. However, these relationships varied. The SFA and PUFA contents in the meat were significantly correlated with dietary SFAs, but the correlation was rather negligible in the case of MUFAs. The fatty acid profile in the meat significantly affected chosen texture parameters and sensory traits, which were most beneficial in the meat with the higher fat content and higher fat saturation index. For example, the higher content of C18:1n-9 positively influenced meat tenderness and lightness, while C18:2n-6 and C18:3n-3 had an undesirable effect on the texture and organoleptic traits of the meat. The aroma and taste were not significantly correlated with the fatty acid profile. The fat content in the meat was inversely correlated with shear force and texture parameters, but positively correlated with tenderness and juiciness.

The influence of the dietary fatty acid profile on the quality of pig meat was confirmed in this research. The saturated dietary fats (beef tallow and coconut oil) improved the meat quality traits. The P:S ratio was decreased but was not below the safe level; however, these observations indicate that there may be limitations in the use of such diets with saturated fat content higher than in that of the present experiment.

## Figures and Tables

**Table 2:** Fatty acid profile in *longissimus* muscle fat.

Item	Group I (Control) SBM + RO	Group II cDDGS + RO	Group III cDDGS + BT	Group IV cDDGS + CO	*p* Value	SEM
C8:0 caprylic	0.08	0.06	0.09	0	-	-
C10:0 capric	0	0	0.27	0.65	-	-
C12:0 lauric	0.42 a	0.35 a	0.45 a	1.01 b	0.008	0.071
C14:0 myristic	1.36 a	1.32 a	1.41 a	2.33 b	0.005	0.111
C16:0 palmitic	20.60 b	18.60 a	21.46 b	22.13 b	0.003	0.402
C16:1 n-7 palmitoleic	2.36	2.39	2.37	2.42	0.993	0.068
C18:0 stearic	12.10	11.88	12.58	12.38	0.365	0.146
C18:1 n-9 oleic	35.35	37.14	36.81	35.80	0.641	0.535
C18:2 n-6 linoleic	16.75 ab	18.12 b	14.93 a	14.02 a	0.042	0.585
ɤ C18:3 n-6 gama-linolenic	0.16	0.13	0.15	0.13	0.501	0.008
C18:3 n-3 alpha-linolenic	1.34 c	1.03 b	0.66 a	0.65 a	0.001	0.074
C20:0 arachidic	0.32	0.28	0.33	0.36	0.551	0.019
C20:4 n-6 arachidonic	6.28	6.21	6.66	5.36	0.706	0.383
C20:5 n-3 EPA	0.54 b	0.32 a	0.22 a	0.22 a	0.005	0.041
C22:0 behenic	0.51 b	0.34 a	0.22 a	0.33 a	0.002	0.031
C22:1 n-9 erucic	0.22 b	0.22 b	0.09 a	0.07 a	0.003	0.021
C22:6 n-3 DHA	0.91 b	0.50 a	0.33 a	0.21 a	0.001	0.077
SFA	35.31 b	32.83 a	36.63 b	39.20 c	0.005	0.612
UFA	63.96 b	66.05 c	62.23 b	58.86 a	0.002	0.691
MUFA	37.94	39.74	39.28	38.28	0.674	0.562
PUFA	26.02 b	26.31 b	22.95 ab	20.58 a	0.042	0.919
PUFA n-6	23.22	24.46	21.74	19.51	0.147	0.804
PUFA n-3	2.80 c	1.85 b	1.21 a	1.07 a	0.001	0.176
PUFA n-6/n-3	8.45 a	13.25 b	18.09 c	18.38 c	0.005	0.360
PUFA:SFA ratio (P:S)	0.74 b	0.80 b	0.63 ab	0.52 a	0.015	0.036
Fat saturation index (S:P)	0.53 ab	0.48 a	0.57 b	0.63 c	0.002	0.015
Iodine value (IV)	65.81 b	68.79 b	61.95 a	59.44 a	0.008	0.961

a, b, c—mean values in the same row with different letters differ significantly at *p* ≤ 0.05. SEM—standard error of mean; SFA—sum of saturated fatty acids; UFA—sum of unsaturated fatty acids; MUFA—sum of monounsaturated fatty acids; PUFA—sum of polyunsaturated fatty acids.

**Table 3:** Meat quality traits (*longissimus* m.)**.**

Item	Group I (Control) SBM + RO	Group II cDDGS + RO	Group III cDDGS + BT	Group IV cDDGS + CO	*p* Value	SEM
Basic chemical analysis, g/kg:						
- dry matter	240.20	238.00	244.90	250.50	0.085	1.924
- protein	236.00 b	228.00 a	227.20 a	233.70 b	0.017	1.250
- fat	9.20 a	10.50 ab	15.90 b	15.60 b	0.045	1.149
Oxidative stability after 4 months (−20 °C):						
TBARS, mg/kg	0.47	0.49	0.47	0.46	0.827	0.013
Acidity:						
- pH 45 min after slaughter	6.48	6.38	6.36	6.35	0.337	0.028
- pH after 24h (+4 °C)	5.57	5.40	5.50	5.53	0.899	0.037
- pH after 4 months (−20 °C)	5.42	5.55	5.50	5.47	0.326	0.024
Water holding capacity:						
WHC index after 24h (+4 °C), cm^2^/g	22.32 b	21.80 ab	18.80 a	18.65 a	0.038	0.603
WHC index after 4 months (−20 °C), cm^2^/g	15.73	15.15	13.99	13.78	0.352	0.440
Drip loss during defrosting, %	13.97	14.96	14.07	13.96	0.874	0.467
Thermal (cooking) drip loss, %	27.19 b	24.53 a	24.09 a	24.63 a	0.042	0.458
Meat color after 24 h (+4 °C):						
- lightness (L)	52.02	52.24	51.72	51.77	0.564	0.564
- saturation in red (a)	17.29	16.45	16.03	16.46	0.060	0.178
- saturation in yellow (b)	2.35	2.21	2.13	2.36	0.808	0.091
- color saturation (C)	17.46	16.60	16.18	16.64	0.062	0.181
Meat color after 4 months (−20 °C):						
- brightness (L*)	50.57	50.00	50.51	48.17	0.749	0.841
- saturation in red (a*)	17.52	17.14	16.80	16.91	0.304	0.146
- saturation in yellow (b*)	3.03	2.81	2.97	3.19	0.852	0.141
- color saturation (C)	17.80	17.38	17.08	17.21	0.299	0.142
Color change during storage, ΔE (4 months −20 °C)	1.77	2.74	2.22	2.05	0.287	0.181

a, b—mean values in the same row with different letters differ significantly at *p* ≤ 0.05; SEM—standard error of mean.

**Table 4:** Shear force and texture profile analysis of meat (*longissimus* m.).

Item	Group I (Control) SBM + RO	Group II cDDGS + RO	Group III cDDGS + BT	Group IV cDDGS + CO	*p* Value	SEM
Shear force, N	63.66	61.12	59.27	57.84	0.859	2.436
Toughness,	193.60	183.02	178.78	166.75	0.708	39.843
Hardness	9.44	7.36	6.35	6.51	0.264	0.631
Springiness	0.72	0.73	0.68	0.69	0.263	0.009
Cohesiveness	0.69 b	0.68 b	0.64 a	0.63 a	0.024	0.010
Chewiness	4.74 b	3.80 ab	2.66 a	2.75 a	0.047	0.339
Resilience	0.31 b	0.32 b	0.28 a	0.27 a	0.019	0.006

a, b—mean values in the same row with different letters differ significantly at *p* ≤ 0.05; SEM—standard error of mean.

**Table 5:** Sensory traits of cooked meat (*longissimus* m.).

Item	Group I (Control) SBM + RO	Group II cDDGS + RO	Group III cDDGS + BT	Group IV cDDGS + CO	*p* Value	SEM
Aroma	4.68	4.80	4.89	4.76	0.165	0.032
Taste	4.78	4.82	4.89	4.82	0.750	0.033
Tenderness	4.50 a	4.83 b	4.87 b	4.87 b	0.027	0.040
Juiciness	4.53 a	4.80 b	4.89 b	4.90 b	0.037	0.039

a, b—mean values in the same row with different letters differ significantly at *p* ≤ 0.05; SEM—standard error of mean.

**Table 6:** Correlation coefficients between groups of fatty acids in meat (*longissimus* m.) and chosen meat quality traits.

Meat Quality Traits		Fatty Acids in Meat												
		Groups of Fatty Acids						The Most Abundant Fatty Acids						Iodine Value
		SFA	MUFA	PUFA	PUFAn-6	PUFAn-3	UFA	C16:1	C18:1	C18:2	C18:3	C16:0	C18:0	
Texture parameters	Shear force	−0.34	−0.18	0.41 ^●^	0.38	0.36	0.38	0.02	−0.20	0.41 ^●^	0.37	−0.21	−0.38	0.41 ^●^
	Toughness	−0.16	−0.03	0.18	0.15	0.30	0.22	0.15	−0.05	0.12	0.36	−0.04	−0.13	0.18
	Hardness	0.02	−0.23	0.14	0.09	0.34	0.01	−0.06	−0.23	0.11	0.33	0.06	0.03	0.07
	Springiness	−0.21	−0.43 ^●^	0.43 ^●^	0.40	0.44 ^●^	0.22	−0.10	−0.46 ^●^	0.41 ^●^	0.42 ^●^	−0.21	−0.03	0.30
	Cohesiveness	−0.48 ^●^	−0.33	0.54 ^●^	0.48 ^●^	0.62 ^●^	0.45 ^●^	−0.30	−0.33	0.58 ^●^	0.55 ^●^	−0.58 ^●^	−0.07	0.56 ^●^
	Chewiness	−0.12	−0.22	0.25	0.18	0.47 ^●^	0.15	−0.08	−0.23	0.25	0.47 ^●^	−0.08	−0.02	0.24
	Resilience	−0.45 ^●^	−0.23	0.46 ^●^	0.40	0.65 ^●^	0.41 ^●^	−0.33	−0.22	0.49 ^●^	0.68 ^●^	−0.55 ^●^	−0.01	0.53 ^●^
Organoleptic parameters	Aroma	0.07	0.09	−0.13	−0.07	−0.37	−0.10	0.01	0.10	−0.05	−0.37	0.08	0.36	−0.08
	Taste	0.09	−0.09	−0.02	0.01	−0.14	−0.10	−0.30	−0.05	−0.03	−0.14	0.04	0.38	−0.09
	Tenderness	0.18	0.37	−0.44 ^●^	−0.33	−0.77 ^●^	−0.28	0.01	0.41 ^●^	−0.35	−0.69 ^●^	0.05	0.37	−0.33
	Juiciness	0.26	0.10	−0.34	−0.22	−0.74 ^●^	−0.35	−0.22	0.15	−0.30	−0.72 ^●^	0.08	0.43 ^●^	−0.41 ^●^
Moisture traits and fat content	Water holding capacity	−0.29	0.35	−0.01	−0.08	0.27	0.27	0.10	0.34	−0.02	0.46	−0.30	−0.07	0.24
	Thermal drip loss	0.07	−0.04	−0.01	−0.12	0.50 ^●^	−0.04	−0.07	−0.04	0.05	0.54	0.15	0.07	0.14
	Fat content	0.69 ^●^	0.33	−0.73 ^●^	−0.70 ^●^	−0.61 ^●^	−0.68 ^●^	0.39	0.33	−0.54 ^●^	−0.56 ^●^	0.73 ^●^	0.43 ^●^	−0.52 ^●^
Color traits	Lightness 24 h, L	0.16	0.54 ^●^	−0.48 ^●^	−0.45	−0.43	−0.19	0.12	0.56 ^●^	−0.52	−0.20	0.11	0.44	−0.32
	Redness 24 h, a	−0.24	−0.23	0.34	0.25	0.65 ^●^	0.27	−0.10	−0.25	0.35	0.57 ^●^	−0.15	−0.44	0.36
	Yellowness 24 h, b	−0.12	−0.30	0.33	0.31	0.19	0.19	−0.42	−0.27	0.05	0.19	−0.15	−0.21	−0.05
	Saturation 24 h, C	−0.24	−0.25	0.35	0.26	0.65 ^●^	0.28	−0.09	−0.27	0.35	0.57 ^●^	−0.15	−0.44	0.35
Meat acidity	pH 45 min	0.12	0.48 ^●^	−0.38	−0.44	0.02	−0.11	0.17	0.49 ^●^	−0.45	0.22	0.14	0.32	−0.19
	pH 24 h	0.17	0.27	−0.25	−0.27	−0.06	−0.11	0.19	0.27	−0.33	−0.02	0.07	0.17	−0.22

^●^ Correlation statistically significant at *p* ≤ 0.05.

## Data Availability

The data supporting reported results are in the possession of the corresponding author.
